# Multiomics Integration and Machine Learning Reveal Colony Stimulating Factor 3 Receptor as a Key Gene and Therapeutic Target in Crohn's Disease

**DOI:** 10.1155/mi/1619237

**Published:** 2025-08-28

**Authors:** Peihong Li, Hongyi Hu, Lujia Yang, Linda Zhong, Boyun Sun, Chaoqun Bao

**Affiliations:** ^1^Department of Gastroenterology, Longhua Hospital, Shanghai University of Traditional Chinese Medicine, Shanghai, China; ^2^School of Biological Sciences, Nanyang Technological University, Singapore, Singapore; ^3^The First Affiliated Hospital of Ningbo University, Ningbo, China

**Keywords:** Crohn's disease, CSF3R, machine learning, Mendelian randomization, molecular structure

## Abstract

Crohn's disease (CD) is a chronic inflammatory disease characterized by complex immune dysregulation in which the identification of key molecular drivers is critical for the advancement of diagnostic and therapeutic approaches. In this study, we integrated transcriptomic data from multiple cohorts and applied three machine learning algorithms—Random forest, support vector machine recursive feature elimination (SVM-RFE), and Least Absolute Shrinkage and Selection Operator (LASSO)—to robustly identify key gene, converging on CSF3R as a top candidate. Mendelian randomization (MR) analysis supported a causal role of CSF3R in CD pathogenesis (OR = 1.400, 95% CI: 1.022–1.917). Enrichment analysis revealed its association with cytokine-receptor interactions and the JAK-STAT pathway. Single-cell RNA sequencing and immune infiltration analyses demonstrated elevated CSF3R expression in neutrophils, implicating it in neutrophil-mediated inflammation. Experimental validation using intestinal biopsies from CD patients and healthy controls (HCs) confirmed significantly upregulated CSF3R expression at both mRNA and protein levels, as shown by quantitative reverse transcription PCR (qPCR), western blot, and immunohistochemistry. Double immunofluorescence further revealed strong colocalization of CSF3R with the neutrophil marker CD66b, supporting its functional association with neutrophil infiltration. Moreover, molecular docking indicated high binding affinity between CSF3R and several therapeutic agents, including methotrexate and aspirin. Diagnostic performance assessments yielded high AUC values (0.823–0.938) across multiple datasets. Collectively, these findings highlight CSF3R as a robust diagnostic gene and promising therapeutic target in CD, offering mechanistic insights and opportunities for precision medicine.

## 1. Introduction

Crohn's disease (CD) is a chronic, relapsing inflammatory bowel disease (IBD) that can affect any part of the gastrointestinal tract, with a preference for the terminal ileum and adjacent colon. It presents a characteristic segmental and skipping pattern of lesions. Clinically, CD manifests with abdominal pain, diarrhea, weight loss, abdominal masses, and extraintestinal symptoms, such as dermatological, oral, and ocular lesions [1]. Over recent decades, the prevalence of CD has been increasing globally, with a particularly rapid rise in Asia, attributed to lifestyle changes and environmental factors associated with economic development [2]. In China, CD predominantly affects middle-aged and young adults, though onset can occur at any age, with an approximately equal male-to-female ratio [3]. Despite not being directly life-threatening, CD imposes substantial psychological and financial burdens due to its chronic course, frequent relapses, and limited curative options. Current understanding of CD pathogenesis suggests a complex interplay of genetic susceptibility, environmental triggers, gut microbiota dysbiosis, and immune system dysregulation. These factors collectively contribute to aberrant immune responses and chronic inflammation, yet the precise mechanisms underlying CD remain unclear. This underscores the urgent need for studies aimed at elucidating disease pathways and identifying reliable molecular markers for early diagnosis and personalized therapeutic interventions.

Colony-stimulating factors (CSFs), particularly granulocyte-macrophage colony-stimulating factor (GM-CSF) and granulocyte CSF (G-CSF), play crucial roles in the pathophysiology and potential treatment of CD. GM-CSF has demonstrated notable anti-inflammatory properties in IBD, including the attenuation of experimental colitis and facilitation of intestinal epithelial repair. These effects are mediated through the suppression of pro-inflammatory cytokines, such as tumor necrosis factor-alpha (TNF-α) and interleukin-1 beta (IL-1β), as well as the promotion of ulcer healing and epithelial regeneration [4]. Clinically, GM-CSF has shown efficacy in managing active CD, particularly in enabling hormone-dependent patients to achieve hormone-free remission [5, 6]. Similarly, G-CSF has been found to exert protective effects in colitis by reducing apoptosis in colonic epithelial cells and mitigating DSS-induced colitis in murine models. These effects may be attributed to its ability to suppress pro-inflammatory cytokine production and enhance anti-inflammatory responses [7]. Clinical studies have indicated that recombinant human G-CSF (rhG-CSF) is both safe and potentially effective in treating active CD [8], with particular efficacy in preventing postoperative relapses [9]. While current research primarily focuses on the therapeutic potential of these growth factors, there is limited exploration of how changes in their receptor expression impact CD pathogenesis. Aberrant expression or mutations in GM-CSF receptor subunits, particularly variants in the CSF2RA and CSF2RB genes, can impair GM-CSF signaling, contributing to increased disease susceptibility and a higher incidence of complications [10]. These findings underscore the need for further investigations into the mechanistic implications of CSF receptor alterations in CD, which may uncover novel therapeutic targets and genes.

Given the heterogeneous nature of CD, there is an urgent need to identify reliable key driver genes as therapeutic targets to improve early diagnosis and enable personalized treatment. High-throughput sequencing technologies provide a robust platform for studying differential gene expression in diseases, enabling the discovery of disease-associated genes and uncovering novel therapeutic targets [11, 12]. Furthermore, Mendelian randomization (MR) offers a powerful framework for investigating causal relationships between genetic variants, gene expression, and disease phenotypes [13]. By integrating expression quantitative trait loci (eQTL) data with disease associations, MR analysis can identify genes with direct causal roles in disease pathogenesis while minimizing confounding factors and reverse causation. Machine learning approaches complement these methods by prioritizing candidate genes and refining target selection. Functional analyses, including protein-protein interaction (PPI) network construction, Gene Ontology (GO) annotation, and Gene Set Enrichment Analysis (GSEA), provide insights into the biological significance of key genes and their roles in disease pathways [14, 15]. scRNA-Seq and drug-target enrichment analyses add further depth, enabling validation of candidate genes in specific cell populations and exploration of potential therapeutic applications through molecular docking.

**Figure 1:**
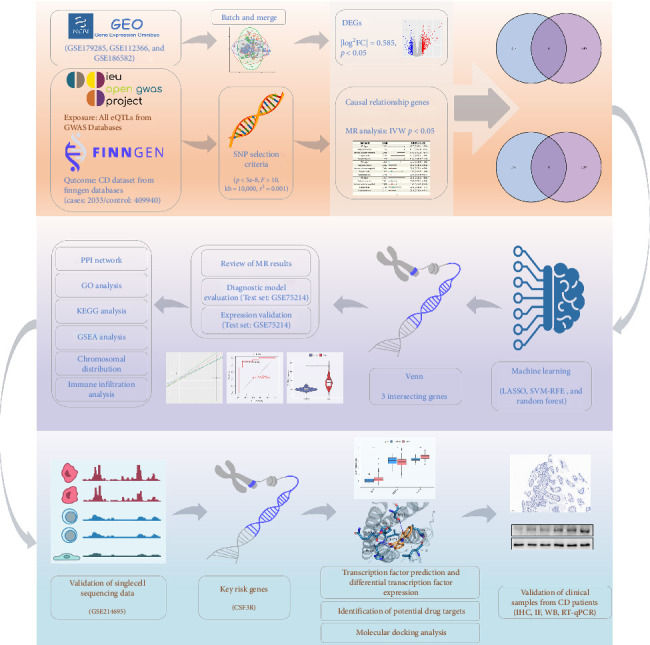
The overall flowchart of this research.

This study integrates these approaches to address critical gaps in the current understanding of CD pathogenesis. First, differentially expressed genes (DEGs) were identified from multiple GEO datasets and cross-referenced with expression quantitative trait locis (eQTLs) identified through MR analysis to pinpoint genes causally linked to CD. Subsequently, ML models were employed to identify key driver genes, which were validated using independent datasets. Functional and pathway analyses were performed to explore the roles of these genes in CD-related molecular networks. Finally, scRNA-Seq data and drug-target enrichment analyses were utilized to validate findings and assess their translational potential. The detailed analytic workflow was shown in Figure 1. By combining multiomics data with advanced computational methods, this study aims to explore the molecular mechanisms of CD in depth, identify key driver genes for early diagnosis, and propose new therapeutic targets.

## 2. Methods

### 2.1. Data Collection

The gene expression data used in this study were obtained from the Gene Expression Omnibus (GEO) database (https://www.ncbi.nlm.nih.gov/geo/), a public repository for high-throughput gene expression and other genomics datasets. The inclusion criteria required datasets to: (1) Provide genome-wide gene expression profiles. (2) Be derived from terminal ileum epithelial tissues during the active phase of CD. (3) Include samples from both CD patients and healthy controls (HCs), with at least 10 samples per group. (4) Exclude datasets involving other diseases, comorbidities, or prior treatment interventions. Based on these criteria, three datasets were selected for the training set: GSE112366, GSE179285, and GSE186582. For validation purposes, three additional datasets were selected as the validation set: GSE75214, GSE193677, and GSE119600. MR analysis was performed using eQTL data from the IEU database and GWAS outcome data from the FinnGen database (K11_CD_STRICT2; *n* cases = 2033, *n* controls = 409,940). Single nucleotide polymorphisms (SNPs) were chosen as instrumental variables (IVs) according to genome-wide significance thresholds and independence assumptions, employing stringent statistical methodologies to pinpoint relevant genes. scRNA-Seq data from GEO (GSE214695) were processed through quality control, Principal component analysis (PCA) integration, and cell type annotation to analyze and validate the expression and distribution of key genes in CD intestinal tissues. All included studies received approval from the appropriate institutional ethics review boards. The basic information of the datasets is detailed in Table 1.

### 2.2. GEO Data Preprocessing and DEGs Analysis

The GSE112366, GSE179285, and GSE186582 datasets were used for DEGs analysis to investigate molecular differences between the CD and control groups. Expression matrices were normalized using the Normalize Between Arrays function in the “*limma*” package to ensure consistency across samples. The merged, normalized dataset underwent batch correction using the ComBat function from the “*sva*” package to mitigate interbatch variability and minimize confounding factors. PCA was performed to evaluate sample distribution, with visualization implemented using the “*ggplot2*” and “*ggpubr*” packages in *R* (version 4.3.1). DEGs were identified through the “*limma*” package, applying thresholds of an adjusted *p*-value < 0.05 and |log2FC| = 0.585 to ensure both statistical robustness and biological relevance. Significant DEGs were visualized using a volcano plot, while heatmaps, generated via the “heatmap” package, depicted distinct expression patterns between the CD and control groups.

### 2.3. MR Analysis to Identify Key Causal Genes

To investigate the causal relationships between gene expression and CD, we utilized eQTL data from the IEU Open GWAS Project (https://gwas.mrcieu.ac.uk/) as the exposure dataset. This dataset comprises 19,942 eQTL datasets derived from European populations. CD-associated GWAS summary statistics (K11_CD_STRICT2) were obtained from the FinnGen database as the outcome data. IVs were selected based on genome-wide significance (*p* < 5× 10^−8^) and stringent linkage disequilibrium parameters (10,000 kb window, *r*^2^ < 0.001). To mitigate weak instrument bias, IV strength was assessed using the *F*-statistic, with a threshold of *F* > 10 considered indicative of robust instruments. MR analysis was conducted using the “*TwoSampleMR*” package in *R* to explore causal relationships between specific gene expressions and CD risk. The primary analytic method was inverse variance weighted (IVW) regression, which assumes no horizontal pleiotropy. To account for potential violations of this assumption, MR-Egger regression was employed to detect directional pleiotropy, while heterogeneity across instruments was evaluated using Cochran's *Q* test. Additionally, the MR-PRESSO method (5000 iterations) was applied to further assess and correct for horizontal pleiotropy. To identify significant genes, we intersected DEGs from transcriptomic analyses with genes exhibiting significant causal effects in MR analysis.

### 2.4. Machine Learning for Candidate Diagnostic Genes Identification

To identify potential diagnostic genes, we applied three machine learning algorithms [16]: Least Absolute Shrinkage and Selection Operator (LASSO) regression, implemented using the “*glmnet*” *R* package, was employed to select the most predictive features by penalizing less informative regression coefficients and reducing overfitting. Support vector machine recursive feature elimination (SVM-RFE), performed with the “e1071” and “caret” *R* packages, optimized feature selection by iteratively eliminating features with the least contribution to model performance. Random forest, an ensemble learning method implemented using the “*RandomForest*” *R* package, enhanced feature selection by combining predictions from multiple decision trees, improving robustness and accuracy. Training datasets (GSE112366, GSE179285, and GSE186582) were preprocessed through normalization, dataset merging, and batch effect correction to ensure data consistency. The final set of key genes was determined by identifying the intersection of features selected by all three algorithms. Validation datasets (GSE75214, GSE193677, and GSE119600) were used to evaluate model performance, with the area under the receiver operating characteristic (ROC) curve (AUC) as the primary metric. AUC results were visualized using heatmaps, and the expression consistency of the identified key genes between the normal and CD groups was tested across all validation datasets to confirm diagnostic relevance.

### 2.5. PPI Network Construction

PPI network was constructed to explore interactions among the identified key genes. The “*STRINGdb*” *R* package was used to retrieve interaction data, while visualization and centrality analyses were performed using the “*igraph*” package. Node importance was ranked by betweenness centrality. Additionally, the GeneMANIA web tool (http://genemania.org/) was utilized to validate and extend the PPI network.

### 2.6. Functional Enrichment Analysis

Functional annotation and pathway enrichment analyses were conducted to investigate the biological significance of the key intersecting genes: GO analysis categorized genes into molecular function (MF), biological process (BP), and cellular component (CC), was performed using the clusterProfiler and enrichGO packages in *R*, with visualization supported by the circlize package. Additionally, Kyoto Encyclopedia of Genes and Genomes (KEGG) pathway analysis was performed to identify higher-order genomic functions, with both analysis and visualization implemented using the clusterProfiler and enrichplot packages.

### 2.7. Immune Infiltration Analysis

The CIBERSORT platform was utilized to identify 22 immune cell subsets [17]. A *p*−value < 0.05 was considered indicative of significant immune cell infiltration. To visualize the findings, the relationship between specific diagnostic markers and infiltrating immune cells was assessed using the ggstatsplot and ggplot2 packages in *R*. A bar plot was generated to show the distribution of immune cells in each group of samples, while a violin plot was used to show differences in immune cell populations between groups.

### 2.8. Validation of Single-Cell Sequencing Analysis

scRNA-seq data from GSE214695 were analyzed to validate and characterize the spatial distribution of candidate genes across different cell populations. Data preprocessing included quality control and normalization using the “*Linnorm*” and “*scater*” *R* packages. Dimensionality reduction was performed using PCA and Uniform Manifold Approximation and Projection (UMAP) in the “*Seurat*” package. Cell clusters were annotated based on marker genes, and functional enrichment analyses were conducted to characterize the biological roles of each cell population. The “*SingleR*” package was used for automated cell type annotation.

### 2.9. Upstream Transcription Factor Analysis and Differential Expression Analysis

The transcription factor regulatory relationships were analyzed using the TRRUST v2 database (www.grnpedia.org/trrust), a freely accessible and manually curated resource that includes 8444 TF-target regulatory interactions covering 800 human transcription factors. Using the “Find Key Regulators for Query Genes” module of TRRUST v2, we identified transcription factors potentially regulating FADSs (fatty acid desaturases). Subsequently, we performed differential expression analysis of these identified transcription factors to compare their expression levels between the test and validation datasets, aiming to assess their consistency and potential biological significance.

### 2.10. Identification of Drug Candidates

To identify potential drug candidates targeting the pathological mechanisms underlying CD, we utilized the Drug Signature Database (DSigDB) accessible through the Enrichr web platform (https://amp.pharm.mssm.edu/enrichr/). DSigDB provides curated information on drug-gene interactions, enabling the identification of compounds with potential therapeutic relevance to the intersecting pathological pathways of key genes and CD.

### 2.11. Molecular Docking

The core active compounds of the identified drugs were retrieved in SDF format from the PubChem database, and the target protein structures were sourced from the Protein Data Bank (PDB). For nonmetal ion proteins, preprocessing included removing water molecules and ligands using PyMOL-2.1.0, followed by hydrogen addition and charge assignment using AutoDock Tools-1.5.6. The structures were then converted to pdbqt format for docking. Docking was performed using vina-2.0 within the PyRx platform, with binding affinities (kcal/mol) calculated to evaluate ligand-receptor stability, where lower affinity values indicated stronger interactions. For metal ion proteins, preprocessing was carried out using Discovery Studio 2019, involving removal of water molecules and ligands, hydrogen addition, completion of incomplete residues, and loop simulation. Energy minimization was performed using the CHARMM force field and the Smart Minimizer algorithm (maximum 2000 steps, RMS gradient 0.01). Docking was conducted at the protein's original ligand-binding site using the CDOCKER semiflexible docking approach, and the conformation with the lowest CDOCKER_ENERGY (kcal/mol) was selected as optimal, with higher-CDOCKER_ENERGY values indicating stronger binding. Docking results were visualized using PyMOL to analyze ligand-receptor interactions.

### 2.12. Histological and Immunofluorescence Staining

Terminal ileum tissue samples were collected from 15 CD patients and 15 matched HCs at Shanghai Tenth People's Hospital, with the study approved by the hospital's Ethics Committee (Approval Number 23KT87). Fresh tissues were fixed overnight in 4% neutral formaldehyde, dehydrated, paraffin-embedded, and sectioned at 5 μm thickness. Sections were dried at 60°C for 1–2 h, deparaffinized in xylene, and rehydrated through a graded ethanol series. Antigen retrieval was performed using citrate buffer under microwave heating (high power: 5 min; medium-low power: 15 min), followed by cooling and PBS washes. For immunohistochemical staining, endogenous peroxidase activity was blocked with 3% H_2_O_2_ (15 min), and nonspecific binding was blocked with 5% BSA (37°C, 15 min). Sections were incubated with primary antibody against CSF3R (Abs113310, Absin, China) at 37°C for 1–2 h or 4°C overnight. After PBS washes, secondary antibody incubation proceeded at 37°C for 45 min, followed by DAB chromogenic development (GK500705, Gene Tech, China) under real-time microscopic monitoring. Hematoxylin counterstaining (30 s–2 min) and differentiation in hydrochloric acid ethanol (3–5 s) were performed before dehydration, drying at 37°C, and mounting with neutral resin. For immunofluorescence staining, following identical tissue processing and blocking procedures, sections were incubated in the dark at room temperature with primary antibodies against CSF3R (1:200, Absin, China) and CD66b (1:200, Affinity Biosciences, China), followed by incubation with fluorophore-conjugated secondary antibodies (1:200, Hua'an Bio, China) for 1 h. Nuclei were counterstained with DAPI (Biyuntian, China) for 5 min before mounting. All images were acquired using a Nikon Eclipse C1 fluorescence microscope. Quantitative analysis of protein expression in immunohistochemical sections was performed using the IHC Image Analysis Toolbox plugin in ImageJ 1.54 m software (National Institutes of Health, USA) according to the manufacturer's protocol (https://imagej.nih.gov/ij/plugins/ihc-toolbox/).

### 2.13. Quantitative Reverse Transcription PCR (qPCR)

Total RNA was extracted from terminal ileal tissue using TRIzol Reagent (Thermo Fisher Scientific, Carlsbad, CA) following the manufacturer's instructions and previous study [18]. The RNA concentration and purity were determined using a UV spectrophotometer (Eppendorf, Germany). Reverse transcription was performed using the Evo M-MLVRT Master Mix (Accurate Biotechnology, catalog number AG11706, Changsha, China), and quantitative real-time PCR (RT-qPCR) was conducted with the SYBR Green Pro Taq HS Premix (Accurate Biotechnology, cat. no. AG11740, Changsha, China) according to the manufacturer's protocol. The RT-qPCR thermocycling conditions were as follows: initial denaturation at 95°C for 30 s, followed by 40 cycles of denaturation at 95°C for 5 s and annealing/extension at 60°C for 30 s. Relative mRNA expression levels were calculated using the 2^−ΔΔCt^ method, with β-actin serving as the internal reference gene. The primer sequences used in this study are listed in Table 2.

### 2.14. Western Blot Analysis

Terminal ileum tissues were homogenized in RIPA lysis buffer (P0013B; Beyotime, Hangzhou, China) supplemented with phosphatase inhibitors using a mechanical homogenizer (S1873; Beyotime). Protein concentrations were determined by the biuret method. Total protein extracted from 1 mg of mucosal tissue was resolved through 10% SDS-polyacrylamide gel electrophoresis under reducing conditions and electrophoretically transferred onto polyvinylidene difluoride membranes (Immobilon-P, 0.45 μm; Millipore). Membranes were blocked with 5% nonfat dry milk in Tris-buffered saline containing 0.1% Tween-20 (TBST) for 1 h at room temperature, followed by overnight incubation at 4°C with primary antibodies against CSF3R (1:1000, abs113310, Absin, China) and ACTIN (1:1000, BM0627, Boster, China). After three 10 min TBST washes, membranes were incubated with horseradish peroxidase-conjugated goat anti-rabbit secondary antibody (1:50,000, HA1001, Hua'an Bio, China) for 1 h at room temperature. Protein bands were visualized using an enhanced chemiluminescence detection system (Immobilon Western; Millipore) according to manufacturer specifications. Chemiluminescent signals were captured using a GBOX Chemi XT4 imaging system (Syngene, Cambridge, UK), with semiquantitative densitometric analysis performed using GeneTools software version 4.3 (Syngene), normalizing CSF3R band intensities to corresponding ACTIN controls.

### 2.15. Statistical Analysis

All statistical analyzes were conducted using *R* (version 4.3.1), SPSS (version 25.0), and GraphPad Prism (version 9.0). Transcriptomic data from GEO datasets underwent differential expression analysis via limma (adjusted *p*  < 0.05, |log2FC| = 0.585). MR used eQTL (IEU) and FinnGen GWAS data, employing IVW as the primary method with MR-Egger/MR-PRESSO for pleiotropy correction (IV criteria: *p* < 5 × 10^−8^, *r*^2^ < 0.001, *F* > 10). Machine learning (LASSO/SVM-RFE/Random Forest) identified key genes via 10-fold cross-validation. Functional enrichment (GO/KEGG/GSEA) and protein interactions (STRINGdb) were analyzed with clusterProfiler (*p*  < 0.05). Immune infiltration (CIBERSORT) and single-cell clustering (Seurat) employed Spearman correlation and UMAP. Molecular docking calculated binding affinities (vina/CDOCKER_ENERGY). Experimental data (IHC/qPCR/WB) were compared using *t*-tests (mean ± SD). A *p*-value < 0.05 was considered statistically significant.

## 3. Results

### 3.1. Identification of Key Genes With Causal and Differential Expression in CD

**Figure 2:**
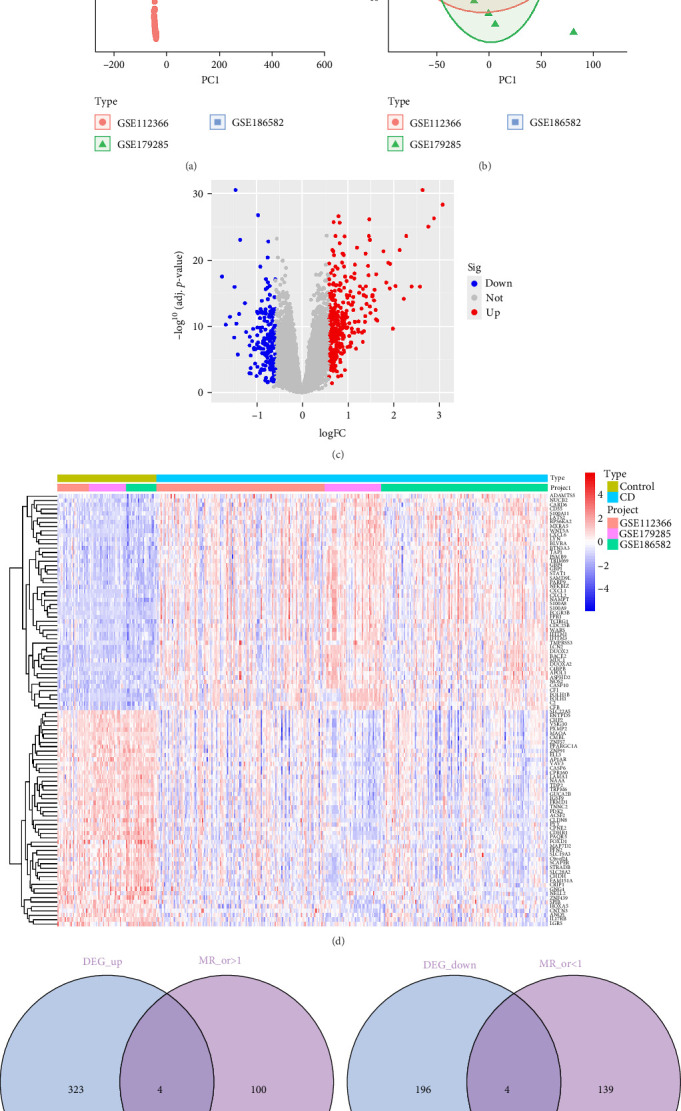
Screening and Visualization of DEGs in CD. (A) PCA before correction, showing dataset separation caused by batch effects from differing experimental conditions. (B) PCA after correction, demonstrating dataset integration with batch effects removed. (C) Heatmap of differential expression analysis. Yellow indicates the control group, blue indicates the experimental group, and shading within each group represents different GEO data sources. (D) Volcano plot of differential expression analysis. Red dots represent upregulated genes, blue dots represent downregulated genes, and white dots represent nondifferential genes. (E) Venn diagram showing the number of upregulated key genes in the overlapping purple region. (F) Venn diagram showing the number of downregulated key genes in the overlapping purple region.

This study utilized three independent datasets (GSE112366, GSE179285 and GSE186582) comprising a total of 326 CD patients and 82 HCs. Following normalization, standardization, and correction for batch effects, the datasets were merged for downstream analyses. PCA was performed to evaluate data distribution before and after merging (Figure 2A,B). DEGs were identified from the merged GEO dataset using the “limma” package in *R*. Genes with a threshold of |log2FC| = 0.585 and *p*  < 0.05 were considered significant. This analysis identified 527 DEGs, including 327 upregulated genes and 200 downregulated genes Supporting Information 1: Table S1. The distribution of DEGs was visualized using a volcano plot (Figure 2C), and their expression profiles were depicted in a heatmap (Figure 2D). For MR analysis of CD, we integrated eQTL data and selected genetic instruments with high variability (*F* > 10). After applying the IVW method, along with exclusion of horizontal pleiotropy and assessment of heterogeneity, 247 genes were identified as having potential causal relationships with CD Supporting Information 1: Table S2. Subsequently, we cross-referenced these 247 causal genes with the DEGs to identify key overlapping genes. This analysis revealed eight key genes: four upregulated (CSF3R, TNFAIP2, CHST15, and PTGDS) and four downregulated (CDKN2B, RETSAT, GPR160, and NAT8B). The intersections of these genes were visualized in Venn diagrams (Figure 2E,F).

### 3.2. Identification of Key Genes in CD Using Machine Learning Techniques

**Figure 3:**
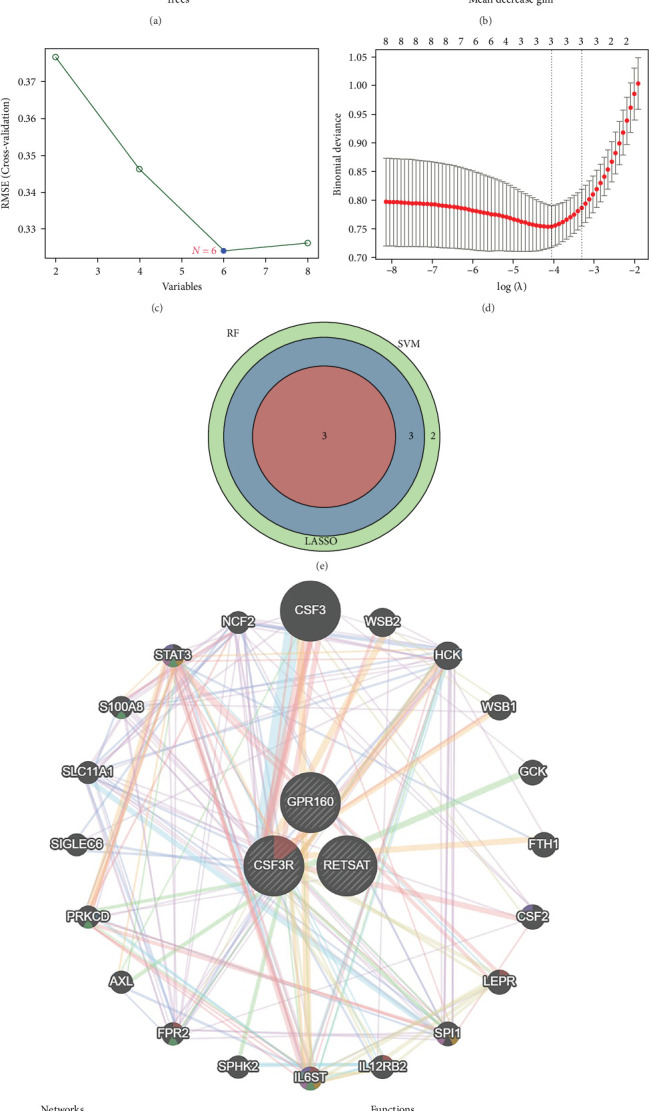
Application of machine learning for key gene identification. (A) Error curve from the random forest algorithm. The horizontal axis represents the number of trees, and the vertical axis represents the cross-validation error. The red line represents the CD group, the green line represents the control group, and the black line represents all samples. (B) Gene importance scores ranked by the random forest algorithm. (C) Cross-validation error plot for the SVM-RFE algorithm. The horizontal axis represents the number of genes retained, and the vertical axis represents the cross-validation error. The optimal number of genes (6) corresponds to the lowest error. (D) Cross-validation error plot for the LASSO regression algorithm. The horizontal axis (log (*λ*)) is the logarithmic scale of the regularization parameter, and the vertical axis represents the cross-validation error. The dashed line (lambda.min) marks the *λ* value where the error is minimized, selecting 3 genes. (E) Venn diagram illustrating intersecting genes across the different machine learning algorithms. (F) PPI network showing the 3 core genes and their interactions with other related genes.

Machine learning approach was employed to identify key genes from the eight crossover candidate genes. Initially, the Random Forest algorithm was used to evaluate feature importance, selecting genes with importance scores greater than two as potential disease-associated signature genes. This process retained all eight candidate genes. The model's performance was optimized by minimizing cross-validation error, which plateaued when the number of trees reached ~500 (Figure 3A,B). Subsequently, the SVM-RFE algorithm was applied to refine the selection. By iteratively removing less informative features, the algorithm identified an optimal subset of six genes: CSF3R, GPR160, RETSAT, CDKN2B, PTGDS, and NAT8B, where the cross-validation error reached its lowest value (Figure 3C). Further reduction was achieved using LASSO regression, which penalizes features with lower contributions. Cross-validation determined the optimal regularization parameter (*λ*), with the log (*λ*) value corresponding to the minimum cross-validation error (*λ*min). This analysis reduced the gene set to three key genes: CSF3R, GPR160, and RETSAT (Figure 3D). To integrate results from all three algorithms, a Venn diagram was constructed, confirming CSF3R, GPR160, and RETSAT as the final key genes (Figure 3E). Further analysis using a PPI network revealed functional associations among these genes, highlighting their pivotal roles in pathways associated with CD (Figure 3F).

### 3.3. MR Results of the Intersected Genes

**Figure 4:**
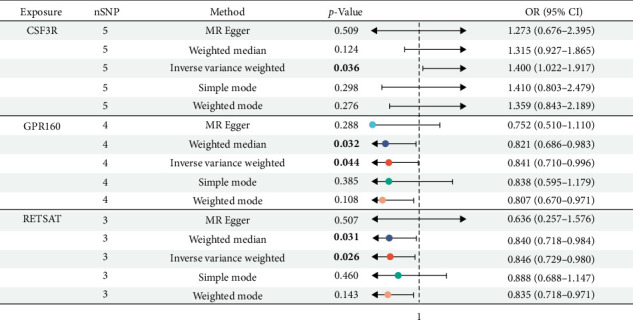
MR results of the three intersected genes.

MR analysis using the IVW method provided evidence of associations between the intersected genes and CD. For the CSF3R gene, nSNP = 5, OR = 1.400 (95% CI: 1.022–1.917), indicating a significant positive association with CD risk. For the GPR160 gene, nSNP = 4, OR = 0.841(95% CI: 0.710–0.996), while for the RETSAT gene, nSNP = 3, OR = 0.846 (95% CI: 0.729–0.980), both suggesting significant negative associations with CD risk. These results indicate that increased expression of CSF3R may contribute to a higher risk of CD, whereas higher expression levels of GPR160 and RETSAT appear to be protective against disease onset. The detailed statistical outcomes are presented in Figure 4 and Supporting Information 1: Table S3.

### 3.4. Diagnostic Evaluation and Validation of Intersected Genes in CD

**Figure 5:**
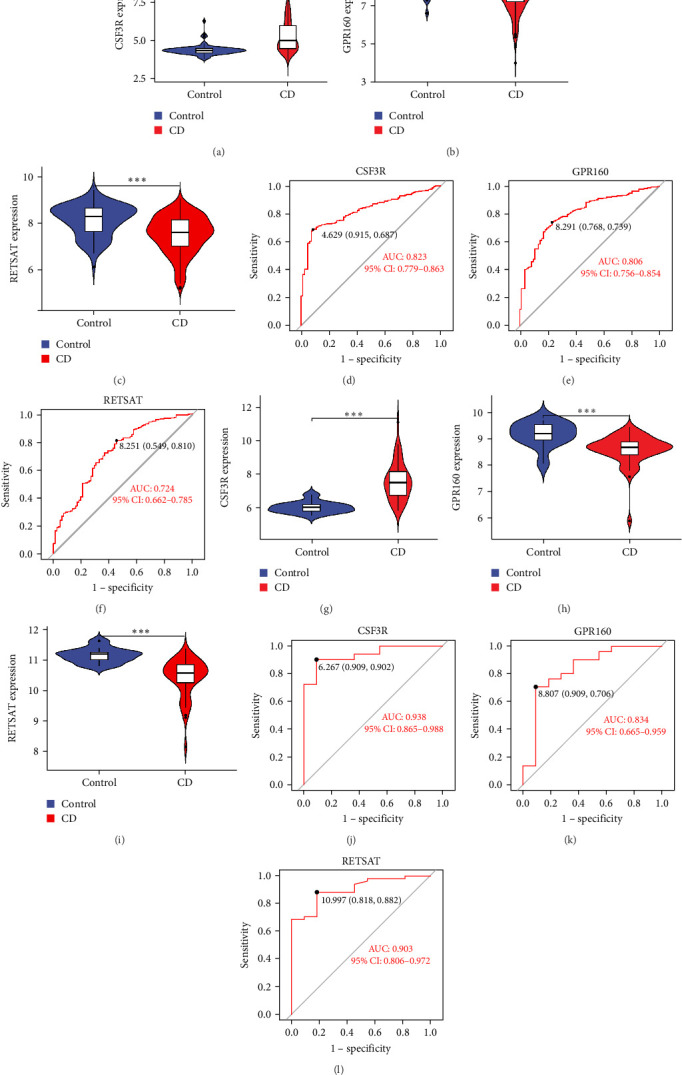
Validated diagnostic potential of intersected genes in cohort data. (A–C) violin plots of CSF3R, GPR160, and RETSAT expression in the test set. (D–F) ROC curves of CSF3R, GPR160, and RETSAT in the test set. (G–I) Violin plots of CSF3R, GPR160, and RETSAT expression in the validation set. (J–L) ROC curves of CSF3R, GPR160, and RETSAT in the validation set. *⁣*^*∗∗∗*^*p* < 0.001.

We performed a differential expression analysis of the intersected genes across groups and visualized the results using violin plots. To evaluate the diagnostic potential of these genes, ROC curve analysis was performed, generating ROC curves and corresponding statistical metrics for each gene. These analyses were rigorously conducted on both the training merged dataset and the external validation dataset (GSE75214). In the training dataset, the expression of CSF3R was significantly upregulated in CD (Figure 5A), while the expressions of GPR160 and RETSAT were significantly downregulated (Figure 5B,C). These findings were consistently replicated in the validation dataset, where CSF3R expression was again significantly elevated (Figure 5G), and GPR160 and RETSAT expressions remained significantly reduced (Figure 5H,I), supporting their potential diagnostic relevance for CD. ROC analysis demonstrated excellent diagnostic performance for all three genes in the training dataset, with CSF3R (AUC = 0.823; 95% CI: 0.779–0.863), GPR160 (AUC = 0.806; 95% CI: 0.756–0.854), and RETSAT (AUC = 0.724; 95% CI: 0.662–0.785) (Figure 5D,E,F and Supporting Information 1: Table S4). This diagnostic capability was further validated in the external dataset (GSE75214), yielding even stronger results: CSF3R (AUC = 0.938; 95% CI: 0.865–0.988), GPR160 (AUC = 0.834; 95% CI: 0.665–0.959), and RETSAT (AUC = 0.903; 95% CI: 0.806–0.972; Figure 5J,K,L and Supporting Information 1: Table S4). In addition, we conducted supplementary validation of the risk gene CSF3R. In the larger tissue sample validation dataset GSE193677, CSF3R demonstrated good diagnostic performance, with an AUC of 0.824 (95% CI: 0.775–0.873; Supporting Information 2: Figure S1), indicating high diagnostic accuracy. In the peripheral blood sample validation dataset GSE119600, CSF3R also demonstrated encouraging diagnostic potential, with an AUC of 0.779 (95% CI: 0.678–0.870; Supporting Information 2: Figure S2). These results highlight the robust diagnostic value and broad applicability of CSF3R, GPR160, and RETSAT as key genes for identifying CD in diverse patient populations.

### 3.5. Chromosomal Distribution and Functional Enrichment Analysis of Intersected Genes

**Figure 6:**
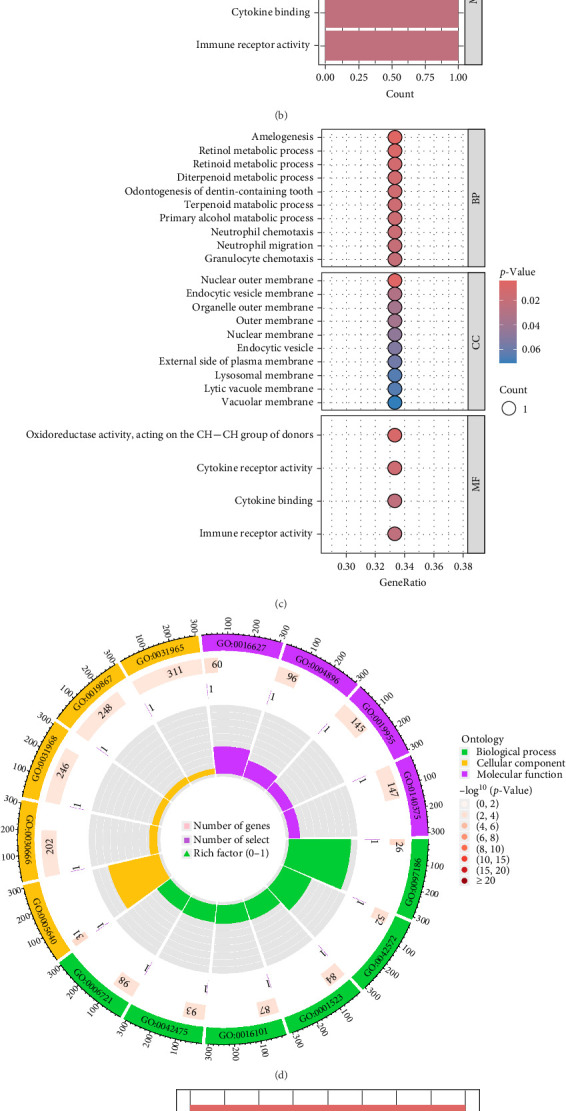
Distribution of intersected genes on chromosomes and their functional enrichment analysis. (A) Distribution of intersected genes on chromosomes. (B) Bar graph of GO analysis, showing the top 10 biological processes in BP, CC, and MF. (C) Bubble chart of GO analysis, with the *x*-axis representing gene proportion and *y*-axis representing enrichment items, bubble size representing the number of genes. (D) Circos plot visualizing the three processes of GO. (E) Bar graph of KEGG, showing the top 3 items. (F) Bubble chart of KEGG, showing the top 3 items, with *x*-axis representing gene proportion and *y*-axis representing enrichment items, bubble size representing the number of genes.

The intersected genes were mapped to their chromosomal locations and visualized using a circos plot, which identified CSF3R on chromosome 1, RETSAT on chromosome 2, and GPR160 on chromosome 3 (Figure 6A). GO enrichment analysis revealed that these genes were involved in several BP, including amelogenesis, retinol metabolic process, and retinoid metabolic process. CC enrichment showed associations with structures, such as the nuclear outer membrane, endocytic vesicle membrane, and organelle outer membrane. MF analysis highlighted roles in oxidoreductase activity acting on the CH─CH group of donors, cytokine receptor activity, and cytokine binding (Figure 6B,C, D and Supporting Information 1: Table S5). KEGG pathway analysis indicated significant enrichment in pathways, such as retinol metabolism, hematopoietic cell lineage, and the JAK-STAT signaling pathway (Figure 6E,F and Supporting Information 1: Table S6).

### 3.6. Gene Expression-Associated Pathway Enrichment in GSEA Analysis

**Figure 7:**
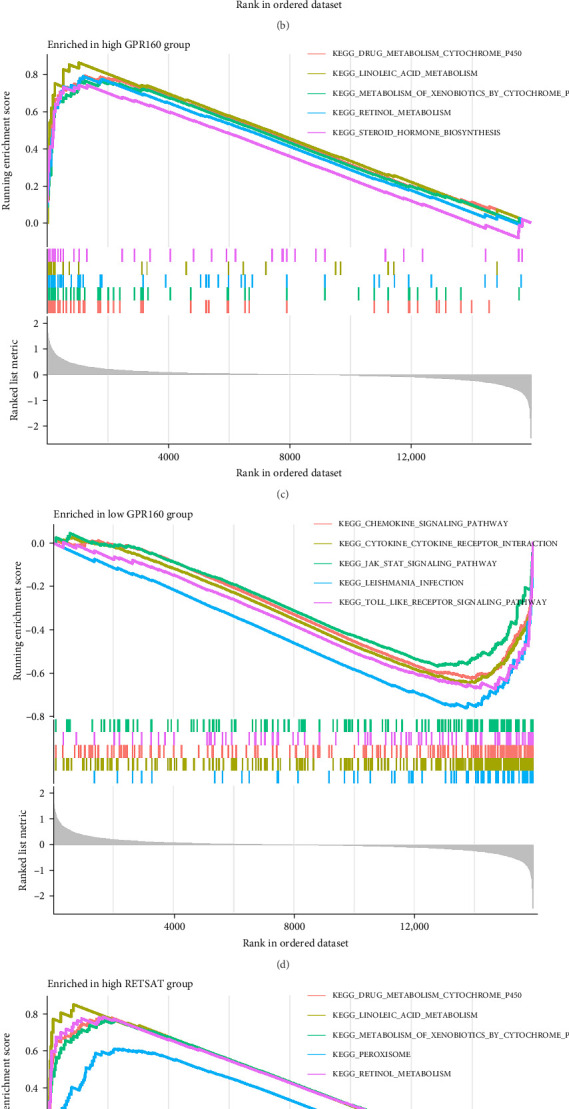
GSEA analysis of intersecting genes. The horizontal axis represents sorted genes, and the vertical axis represents enrichment scores. Different colors denote distinct functions or pathways, with the curve's peak indicating pathway enrichment in high- or low-expression groups. (A) Pathway enrichment in the CSF3R high-expression group. (B) Pathway enrichment in the CSF3R low-expression group. (C) Pathway enrichment in the GPR160 high-expression group. (D) Pathway enrichment in the GPR160 low-expression group. (E) Pathway enrichment in the RETSAT high-expression group. (F) Pathway enrichment in the RETSAT low-expression group.

In the GSEA analysis, the high and low expression groups of CSF3R, GPR160, and RETSAT were defined based on a median-based classification. Samples with expression levels above or equal to the median were designated as the high expression group, while those below the median were assigned to the low expression group. This method ensures equal sample distribution, minimizes subjectivity, and enhances reproducibility by being less sensitive to outliers. For CSF3R, the high-expression group demonstrated significant enrichment in pathways, such as the chemokine signaling pathway and cytokine-cytokine receptor interaction (Figure 7A). In contrast, the low-expression group was enriched in pathways like drug metabolism-cytochrome P450 and linoleic acid metabolism (Figure 7B). Similarly, the high-expression groups of GPR160 and RETSAT exhibited enrichment in pathways related to drug metabolism-cytochrome P450 and linoleic acid metabolism (Figure 7C,E), while their low-expression groups were enriched in the Chemokine signaling pathway and Cytokine-cytokine receptor interaction, among other pathways (Figure 7D,F). Further details can be found in Supporting Information 1: Table S7.

### 3.7. Immune Infiltration and Intersecting Gene Correlations in CD

**Figure 8:**
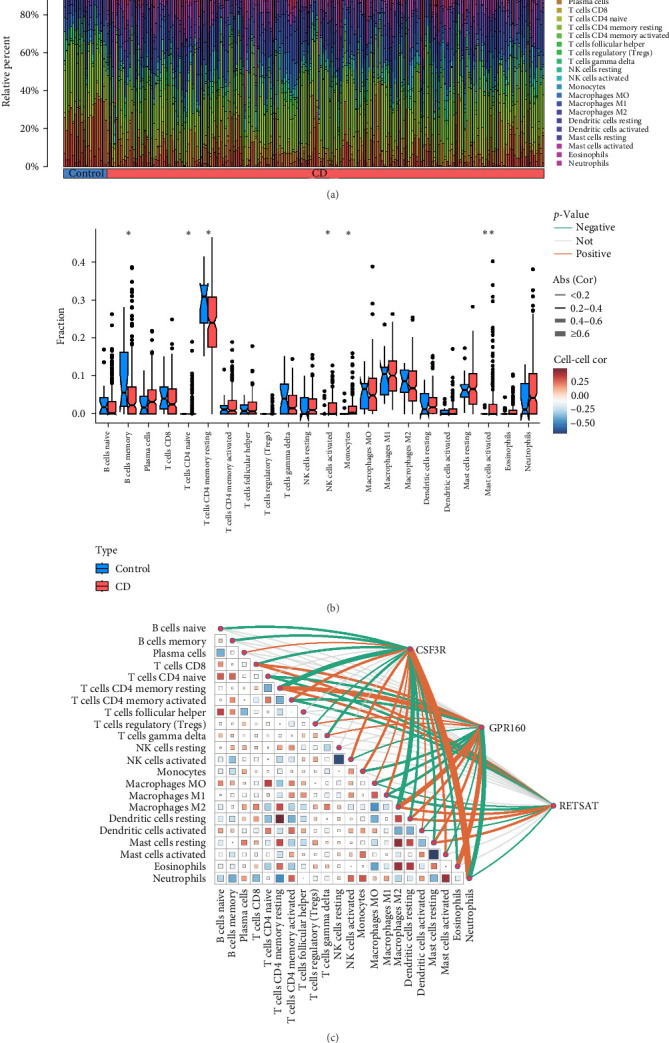
Immune infiltration-related analysis, showing the infiltration of 22 types of immune cells in CD group and healthy population samples. (A) The vertical axis represents the relative amount of immune cells, with different colors indicating different immune cells, and the horizontal axis shows blue for the control group and red for the CD group. (B) The violin plot indicates differences in immune cell infiltration between the control and CD groups. (C) Immune cell interaction heatmap, with horizontal and vertical axes representing various immune cells. Red dots indicate positive correlation, blue dots indicate negative correlation, and size represents the degree of correlation. The top right corner shows the interaction of genes with corresponding immune cells, with green lines indicating negative correlation and yellow lines indicating positive correlation. The thickness of the lines represents the magnitude of the correlation coefficient; thicker lines indicate a larger correlation coefficient. *⁣*^*∗*^*p<*0.05, *⁣*^*∗∗*^*p<*0.01.

To further explore the diagnostic and therapeutic target role of the risk gene CSF3R in CD, we conducted immune infiltration analysis to investigate the immune cell composition and its correlation with CSF3R expression. Using the CIBERSORT algorithm, we characterized the immune cell profiles in CD samples and HCs, visualizing the infiltration levels of 22 immune cell types through heatmaps and violin plots (Figure 8A,B). Our analysis revealed significant differences in immune cell proportions between CD and normal samples, with CD samples showing an increased proportion of naive CD4 + *T* cells, activated NK cells, monocytes, and activated mast cells, and a decreased proportion of memory *B* cells and resting memory CD4 + *T* cells. Notably, CSF3R expression was found to be predominantly associated with neutrophil infiltration, a key feature of CD pathogenesis. This correlation is highly relevant to CSF3R's as potential a diagnostic biomarker and therapeutic target, as it reflects the gene's role in regulating neutrophil activation and migration, which are critical in the inflammatory processes of CD. High CSF3R expression in neutrophils correlates with increased inflammatory markers and may indicate disease flare-ups, thus enhancing its diagnostic utility. Furthermore, the analysis of correlations between the three intersecting genes and immune cell types (Figure 8C) reinforces the concept that CSF3R is involved in multiple immune pathways, suggesting that targeting CSF3R could modulate various immune cell populations implicated in CD. This analysis provides essential insights into the immune dysregulation in CD and supports the relevance of CSF3R in the context of the disease.

### 3.8. Cell Type-Specific Enrichment of Key Genes in CD Colon Tissues

**Figure 9:**
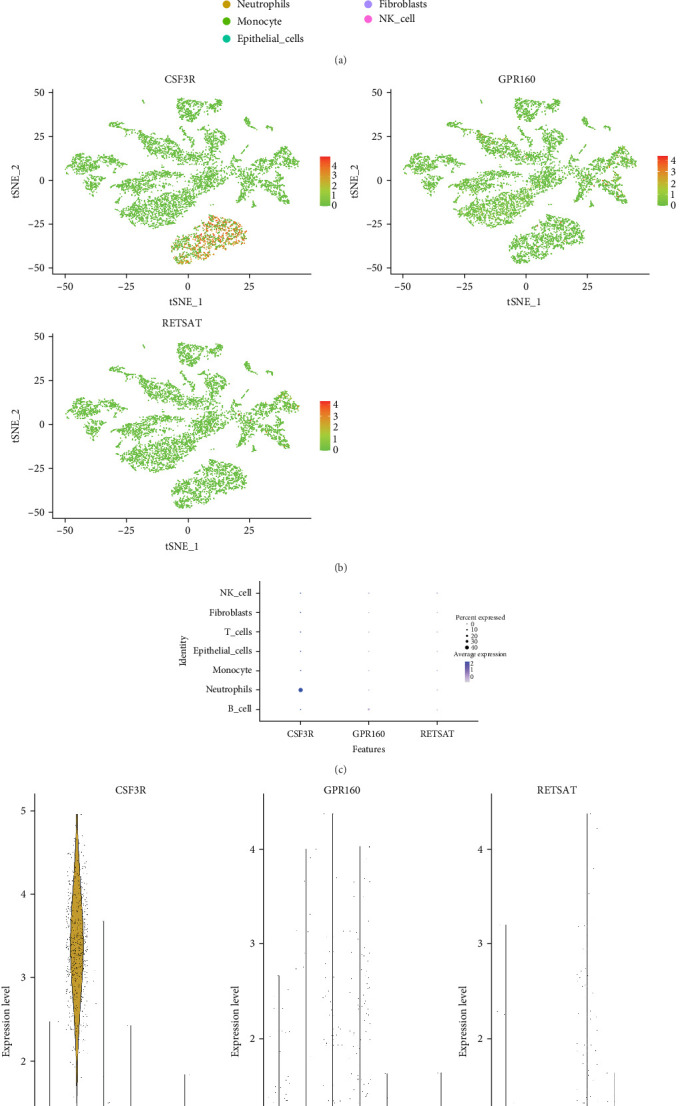
Single-cell sequencing identification of key gene distribution in CD cells. (A) Cells were divided into seven clusters based on cell-specific marker gene expression, and the clustering results were visualized using *t*-SNE. (B) Scatter plot showing the distribution and expression of key genes in different cell types. (C) Bubble plot illustrating the expression of key genes in different cell types. (D) Violin plot of the expression of key genes in different cell types.

To investigate the cell type-specific enrichment of key genes in the colon tissues of CD patients, we analyzed scRNA-Seq data from the CD dataset GSE214695. Dimensionality reduction techniques, including *t*-SNE, were applied to visualize the results, allowing for the clustering of cells into distinct groups based on marker gene expression. This analysis identified seven cell types: *B* cells, neutrophils, monocytes, epithelial cells, *T* cells, fibroblasts, and NK cells (Figure 9A,B). Further analysis revealed that CSF3R expression was significantly enriched in neutrophils, as shown in Figure 9C,D.

### 3.9. Transcription Factor Prediction and Differential Transcription Factor Expression of CSF3R

**Figure 10:**
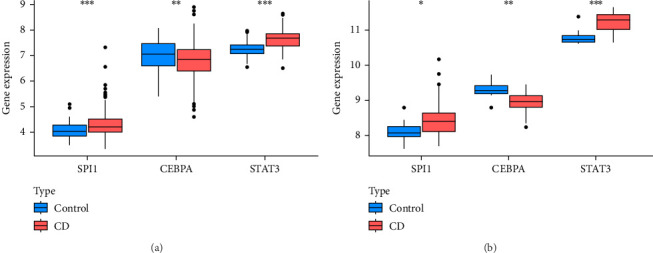
Differential expression of transcription factors regulating CSF3R. (A) expression profiles in the training dataset. (B) expression profiles in the validation dataset (GSE75214).

To further explore the regulatory mechanism of the risk gene CSF3R, we utilized the TRRUST v2 database to predict its upstream transcription factors. The analysis identified SPI1 (*p*=0.010), CEBPA (*p*=0.017), and STAT3 (*p*=0.036) as significant regulators. These transcription factors are known to play critical roles in immune response modulation, hematopoiesis, and inflammation, all of which are relevant to the pathogenesis of CD. Further differential expression analysis revealed distinct patterns of transcription factor activity between CD and control groups. In both the test and validation datasets, SPI1 and STAT3 were significantly upregulated in the CD group, indicating their potential involvement in the disease's pro-inflammatory signaling pathways. In contrast, CEBPA expression was downregulated, suggesting a possible impairment in pathways associated with anti-inflammatory or differentiation processes, as shown in Figure 10A,B.

### 3.10. Identification of Potential Drug Targets and Molecular Docking Analysis

**Figure 11:**
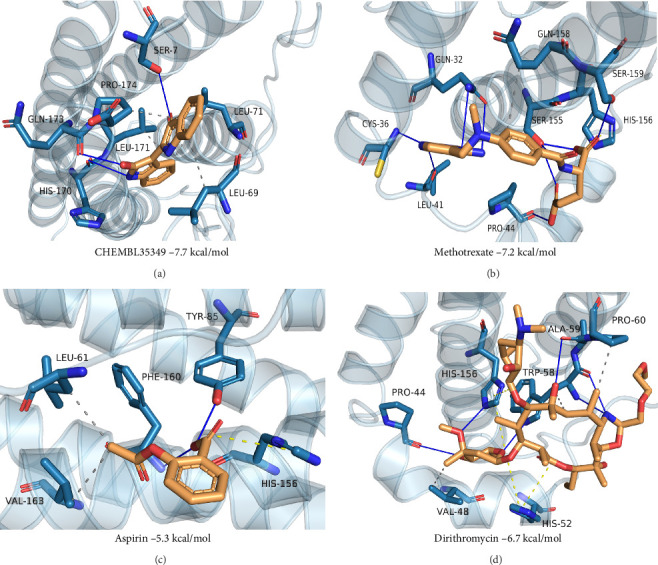
Molecular docking analysis of CSF3R protein with potential drug candidates. (A) CHEMBL35349 exhibited strong binding affinity (*ΔG* = −7.7 kcal/mol) through hydrophobic interactions with LEU69, LEU71, LEU171, and GLN173, and hydrogen bonds with SER7, HIS170, and GLN173. (B) Methotrexate showed robust binding (*ΔG* = −7.2 kcal/mol) via hydrophobic contacts with GLN158, hydrogen bonds with GLN32, CYS36, LEU41, PRO44, SER155, SER159, and a salt bridge with HIS156. (C) Aspirin demonstrated moderate binding (*ΔG* = −5.3 kcal/mol) through hydrophobic interactions with LEU61 and VAL163, hydrogen bonds with TYR85 and PHE160, and a salt bridge with HIS156. (D) Dirithromycin displayed favorable binding (*ΔG* = −6.7 kcal/mol) via hydrophobic interactions with VAL48, TRP58, and PRO60, hydrogen bonds with PRO44, TRP58, ALA59, and HIS156, and salt bridges with HIS52 and HIS156. Key interactions include hydrogen bonds (blue dashed lines), hydrophobic contacts (orange), and salt bridges (yellow dashed lines).

To explore potential therapeutic strategies targeting CSF3R, a combination of drug target identification and molecular docking analysis was employed. Using the DSigDB drug database via Enrichr, four potential drug candidates with significant relevance (*p*  < 0.05) were identified: CHEMBL35349, dirithromycin, aspirin, and methotrexate Supporting Information 1: Table S8. Molecular docking analysis was then performed to assess the binding affinities and interaction mechanisms of these compounds with the CSF3R protein. CHEMBL35349 and methotrexate demonstrated binding energies below −7.0 kcal/mol, indicative of strong binding affinity (Figure 11A,B). Aspirin and dirithromycin displayed binding energies below −5.0 kcal/mol, signifying good binding affinity (Figure 11C,D). Further investigation into the interaction patterns revealed specific molecular engagements. CHEMBL35349 formed hydrophobic interactions with LEU69, LEU71, LEU171, and GLN173, as well as hydrogen bonds with SER7, HIS170, and GLN173, indicating a robust binding profile. Methotrexate exhibited hydrophobic interactions with GLN158 and hydrogen bonds with GLN32, CYS36, LEU41, PRO44, SER155, and SER159, along with a salt bridge with HIS156, suggesting strong structural compatibility. Aspirin interacted with LEU61 and VAL163 through hydrophobic interactions, formed hydrogen bonds with TYR85 and PHE160, and created a salt bridge with HIS156. Dirithromycin established hydrophobic interactions with VAL48, TRP58, and PRO60, hydrogen bonds with PRO44, TRP58, ALA59, and HIS156, and salt bridges with HIS52 and HIS156. Among the identified compounds, CHEMBL35349 and methotrexate emerged as the most promising candidates due to their strong binding affinities and diverse interaction profiles.

### 3.11. Experiment Validation Results

**Figure 12:**
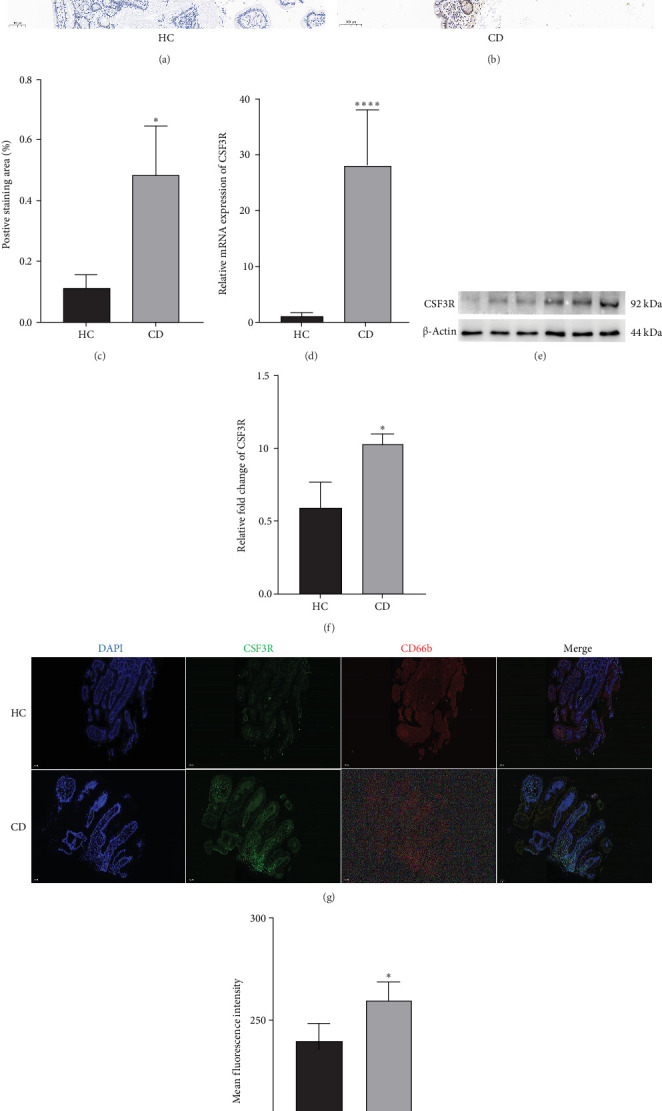
Expression of CSF3R in HC and CD groups and fluorescence colocalization of CSF3R with neutrophils. (A) and (B) show representative immunohistochemical images of the HC and CD groups, respectively (*n* = 15 × 100). (C) shows the statistical analysis of the positive staining area of CSF3R immunohistochemistry (*n* = 15). (D) shows the relative expression levels of CSF3R mRNA detected by RT-PCR (*n* = 15). (E) and (F) present CSF3R protein Western blot images and their relative expression level statistics, respectively (*n* = 15). (G) shows the fluorescence colocalization images of CSF3R (green) and neutrophil marker CD66b (red) in the HC and CD groups (DAPI-stained nuclei, blue), and (H) presents the statistics of the average fluorescence intensity in the coexpression regions (*n* = 15). Data are expressed as mean ± SD. Statistical analysis was performed using *t*-test. *⁣*^*∗*^*p*  < 0.05, *⁣*^*∗∗∗∗*^*p*  < 0.0001.

To further investigate the role of CSF3R in CD, this study conducted experimental validation analyses on intestinal biopsy tissues from CD patients and HCs. Immunohistochemical results showed that the crypt structures in the intestinal tissues of CD patients were disrupted, with a significant infiltration of inflammatory cells into the mucosal layer. Compared with the healthy control (HC) group, the positive staining area of CSF3R in the colonic tissue of CD patients was markedly enlarged, suggesting that CSF3R exhibits broader expression in the intestinal tissue of CD patients. The high expression of CSF3R may be closely associated with these pathological changes and may play a key role in the recruitment and activation of inflammatory cells (Figure 12A,B,–C). RT-PCR analysis revealed that the relative mRNA expression level of CSF3R in the CD group was significantly higher than that in the HC group (Figure 12D), indicating that CSF3R is upregulated at the transcriptional level. Western blot experiments further confirmed this trend, with CSF3R protein expression levels in the CD group also significantly higher than in the HC group (Figure 12E,F), indicating that CSF3R exhibits high expression throughout the entire process from gene transcription to protein translation in the intestinal tissue of CD patients. Double immunofluorescence experiments revealed a positive correlation between CSF3R and the neutrophil marker CD66b (Figure 12G,H). In the intestinal tissue of CD patients, the fluorescence intensity of the coexpression region of CSF3R and CD66b was significantly higher than that in the HC group, suggesting that CSF3R may be associated with neutrophil aggregation and activation. Given the critical role of neutrophils in intestinal inflammation, the high expression of CSF3R may play an important role in promoting neutrophil infiltration and exacerbating intestinal inflammatory responses, suggesting that CSF3R may play a significant role in the pathogenesis of CD.

## 4. Discussion

In this study, eight candidate genes were identified by integrating multiple transcriptomic datasets using differential expression and MR analysis. They were then refined into three key genes using machine learning methods including Random Forest, SVM-RFE, and LASSO regression: CSF3R, GPR160, and RETSAT, whose biological roles are consistent with key features of CD pathogenesis. CSF3R encodes a receptor critical for neutrophil activation, and its expression is upregulated in CD, particularly in neutrophils. This finding underscores its association with the immune dysregulation and neutrophil infiltration that are hallmarks of CD. In contrast, both GPR160 and RETSAT were downregulated in CD and associated with immune regulation and lipid metabolism, respectively. Their altered expression levels may contribute to impaired anti-inflammatory responses and metabolic disturbances in CD patients. Although GPR160 and RETSAT were also identified as key genes, their roles seem to be different from CSF3R. Both genes were significantly downregulated in CD patients and showed a protective correlation with CD risk (GPR160 OR = 0.841; RETSAT OR = 0.846). Functional analysis linked these genes to metabolic and anti-inflammatory pathways, suggesting a role in maintaining intestinal homeostasis. Their inclusion in diagnostic portfolios could improve the robustness of gene-based disease detection. However, their exact mechanistic role in CD remains to be elucidated.

The important finding of this study is to reveal that CSF3R is significantly upregulated in CD patients and positively correlated with the risk of the disease (OR = 1.400, 95% CI: 1.022–1.917). This suggests that CSF3R may play a pivotal role in driving the inflammatory processes underlying CD. CSF3R encodes the G-CSF receptor (G-CSFR), a critical regulator of neutrophil development and activation. Although previous studies on the pathogenesis of CD have focused on GM-CSF and its immunoregulatory function, the relationship between G-CSF and CD has gradually revealed its complexity and duality. On the one hand, G-CSF deficiency has been shown to suppress ileitis in the SHIP-1^−/−^ mouse model [19], and patients with IBD have significantly elevated levels of G-CSF in the pre-relapse phase of the disease [20], suggesting that it may serve as a risk factor for CD relapse. On the other hand, G-CSF shows beneficial effects in alleviating intestinal inflammation by enhancing tissue migration of neutrophils, improving immunodeficiency and protecting the intestinal barrier [7, 8, 21, 22]; for example,rhG-CSF has been shown to significantly reduce epithelial cell apoptosis and inflammatory responses in animal models [7], with clinical studies confirming its safety and therapeutic potential [8]. Similarly, CSF3R plays a dual role in CD and colitis-associated colorectal cancer (CAC). In CD, CSF3R enhances mucosal immune and inflammatory regulation by promoting neutrophil differentiation and IL-17 signaling, which correlates with the activity of the antimicrobial peptide S100A9 and the IL-17 pathway [23]; whereas, in CAC, CSF3R signaling maintains neutrophil function and protects against bacterial invasion, epithelial repair, and IL-22/IL-23 production, and its absence exacerbates colitis and CAC susceptibility [24, 25]. In addition, functional enrichment analysis showed that CSF3R is involved in key pathways, such as cytokine-receptor interactions and JAK-STAT signaling, which is in line with previous studies, where STAT3 activation has been shown to exacerbate the DSS-induced UC model in IBD, and JAK/STAT signaling has been associated with the progression of colitis [26–28], and the role of JAK inhibitors, such as tofacitibib, in the treatment of ulcerative colitis (UC) as evidenced by the clinical efficacy of JAK inhibitors, such as tofacitib [29]. scRNA-Seq revealed that CSF3R is predominantly expressed in neutrophils, which have dual roles in tissue protection and destruction, and its aberrant activation may exacerbate tissue damage and perpetuate the inflammatory cycle. Immune infiltration analysis further confirmed significant immune cell differences between CD patients and HCs, including an increase in initial CD4 + *T* cells, activated NK cells, and mast cells, accompanied by a decrease in memory *B* cells. The strong correlation between key genes and immune cells highlights their role in the regulation of immune responses in CD. To substantiate these findings, we conducted experimental validation in terminal ileal biopsies from CD patients and HCs. CSF3R expression was consistently elevated at both the mRNA and protein levels, as shown by qPCR, Western blot, and immunohistochemistry. Double immunofluorescence staining also revealed colocalization of CSF3R with the neutrophil marker CD66b, supporting its involvement in neutrophil recruitment and activation in inflamed tissue. These findings were further supported by GO, KEGG pathway and GSEA, while subject work characterization (ROC) analysis validated the diagnostic potential of CSF3R, with an AUC of 0.823 in the training dataset and 0.938 in the external validation dataset. Importantly, CSF3R also demonstrated strong diagnostic performance across multiple datasets. In serum-based transcriptomic datasets, CSF3R achieved an AUC of 0.779, which is comparable to or better than widely used markers, such as C-reactive protein (CRP: AUC ~ 0.75–0.81) [30]. Compared with fecal biomarkers like S100A9, which reflect local epithelial inflammation, CSF3R represents a systemic immune activation marker with more direct involvement in immune cell recruitment. Notably, previous studies in pediatric CD patients have also identified CSF3R as a significantly upregulated gene associated with the IL-17 signaling axis [23], reinforcing its potential as a disease-relevant biomarker. The mechanistic distinction between fecal and blood-based biomarkers suggests that they may offer complementary diagnostic value. Given its immunological specificity and the ease of detection from blood samples, CSF3R holds promise as a noninvasive biomarker with potential utility in early diagnosis, disease monitoring, and therapeutic stratification.

Comprehensive analysis of transcriptome regulators of CSF3R showed that SPI1, CEBPA and STAT3 are key upstream transcription factors of CSF3R. In CD, SPI1 and STAT3 were upregulated, which may be involved in the pathogenesis of CD by promoting inflammatory response and immune cell differentiation; on the contrary, the downregulation of CEBPA suggested that the anti-inflammatory mechanism was impaired, which further exacerbated the inflammation response. Together, these findings reveal the complexity of upstream transcriptional regulation of CSF3R and its potential contribution to the pathogenesis of CD SPI1 (also known as PU.1) is an important transcription factor of the ETS family, which plays a central role in myeloid differentiation and is particularly critical for the development and maturation of neutrophils. It has been shown that aberrant expression of SPI1 is closely associated with mutations in the ELANE gene, which encodes elastase, and that mutations in this gene often lead to severe congenital neutropenia (SCN), which is characterized by impaired differentiation and functional defects of neutrophils [31]. CSF3R is a key regulatory molecule for neutrophil survival, proliferation, and differentiation, and its signaling pathway is critical for the maintenance of neutrophil homeostasis. Its signaling pathway is essential for the maintenance of neutrophil homeostasis. Through the activation of CSF3R, neutrophils are able to proliferate and accumulate rapidly under stress, while exhibiting an anti-apoptotic phenotype [32, 33]. These studies suggest that there may be a synergistic role between SPI1 and CSF3R in the differentiation and functional regulation of neutrophils. SPI1 may regulate the strength and duration of the G-CSF signaling pathway by directly or indirectly regulating the transcription of the CSF3R gene, thereby affecting neutrophil maturation and function. However, the direct association between SPI1 and CSF3R and its mechanism of action need to be further investigated. CEBPA expression is significantly downregulated in CD, which may disrupt the anti-inflammatory regulatory mechanism in myeloid differentiation. It has been shown that CEBPA mutations can hinder the normal differentiation of myeloid cells by inhibiting the activity of CSF3R signaling pathway-related enhancers, thereby promoting the proliferation and survival of leukemic cells [34, 35]. This aberrant regulatory mechanism suggests that in CD, downregulation of CEBPA may lead to impaired differentiation of anti-inflammatory cells, exacerbating the inflammatory response and further affecting immune homeostasis. Most studies have reported that STAT3 is an important downstream effector molecule of CSF3R signaling [36–40], with its role in neutrophil generation and various pathological conditions being extensively investigated. G-CSF activates the JAK2/STAT3 signaling pathway through binding to CSF3R, thereby facilitating the generation and release of neutrophils [36, 37]. In inflammatory diseases, the overactivation of STAT3 can influence disease progression by amplifying the inflammatory effects of neutrophils. For instance, in graft-versus-host disease, SOCS1 regulates the inflammatory response by suppressing *T*-cell activation via inhibition of the CSF3R/JAK2/STAT3 signaling pathway [38]. Similarly, in metabolic diseases, such as nonalcoholic fatty liver disease, STAT3 has been implicated in immune cell infiltration and inflammatory regulation through modulation of the GCSFR-SOCS3-JAK-STAT3 signaling pathway [40]. Interestingly, our study suggests that STAT3 may also act as a critical upstream effector of CSF3R signaling, contrasting with the predominant view of STAT3 as a downstream effector. This finding implies a potential negative feedback mechanism through which STAT3 modulates CSF3R signal transduction. These observations collectively highlight the complex and multifaceted roles of STAT3 within the CSF3R signaling pathway, particularly in inflammatory diseases.

Current therapeutic strategies for CD primarily involve a combination of anti-inflammatory drugs, immunomodulators, and pathway-specific biologics. These include immunosuppressive agents (e.g., azathioprine and methotrexate), anti-inflammatory agents (e.g., salazosulfapyridine and glucocorticoids), biologics (e.g., anti-TNF-α antibodies and anti-IL-12/23 antibodies), and targeted small-molecule agents (e.g., Janus kinase inhibitors). Despite significant clinical advancements, a subset of patients continues to exhibit poor responses to existing treatment regimens or experience considerable side effects, underscoring the urgent need for novel therapeutic targets and innovative drugs. Drug target identification and molecular docking analyses have provided valuable insights into potential therapeutic strategies for CD. In this study, CSF3R (G-CSF receptor) was identified as a key risk gene for CD and a promising target for drug development. Molecular docking analysis demonstrated strong binding affinities and significant interaction profiles between CSF3R and certain compounds, including CHEMBL35349 and methotrexate. These findings suggest that such compounds may exert therapeutic effects by modulating the CSF3R-associated signaling pathway, which is closely linked to neutrophil activity and inflammatory responses. Methotrexate has been widely used in the treatment of CD as one of the immunosuppressive agents recommended by clinical guidelines [41]. Molecular docking analysis showed that methotrexate exhibited a strong binding energy ( < −7.0 kcal/mol) by forming multiple hydrogen bonds and salt bridges with CSF3R protein. This finding suggests that methotrexate's action may not be limited to inhibition of dihydrofolate reductase, but may exert additional immunomodulatory functions by targeting CSF3R-related signaling pathways and modulating neutrophil-mediated inflammatory responses. This mechanism provides a new theoretical basis for optimizing the clinical use of methotrexate. In addition, CHEMBL35349 was identified as a new candidate compound that exhibits strong binding ability and abundant molecular interaction patterns, suggesting its potential to specifically modulate the CSF3R signaling pathway. Although its specific pharmacological mechanism needs to be further investigated, as a candidate for the development of novel targeted drugs, it may provide a novel mechanism of action for the treatment of CD. Meanwhile, aspirin and dirithromycin also showed moderate binding affinity in this study, despite their limited potential as directly targeted drugs. Aspirin exerts its anti-inflammatory effects by inhibiting cyclooxygenase (COX), and its interaction with CSF3R suggests that there may be a potential secondary mechanism to modulate CD-specific inflammatory pathways. However, the use of nonsteroidal anti-inflammatory drugs (NSAIDs) like aspirin in IBD remains controversial due to conflicting evidence [42]. Some studies have reported an association between NSAID use and an increased frequency of clinical relapses in quiescent IBD [43, 44], while others suggest potential protective effects, including reduced risks of death, sepsis, and shock in IBD patients [45]. Additionally, some evidence supports the potential of NSAIDs as therapeutic agents for IBD under specific conditions [46]. Nonetheless, the systemic effects of aspirin, particularly its gastrointestinal toxicity, present significant challenges to its clinical application without structural optimization. Dirithromycin is a macrolide antibiotic that has demonstrated immunomodulatory properties in other inflammatory diseases. Its interaction with CSF3R may reflect the prospect of new applications beyond antimicrobial action and warrants further exploration of its potential role in modulating CD-related innate immune responses. Taken together, these findings emphasize the potential for the development of CSF3R-specific inhibitors or modulators, which are capable of attenuating neutrophil-mediated inflammation and provide new directions and strategies for the treatment of CD.

While this study provides valuable insights, it has some limitations. The integration of multiple datasets, while robust, may still be affected by cross-platform technical variability. Despite validation of the findings using independent datasets and scRNA-Seq data, further functional studies are needed to confirm the role of CSF3R and its associated pathways in vitro and in vivo. In addition, the identified therapeutic candidates require further experimental validation to assess their efficacy and safety in the clinical setting.

## 5. Conclusion

In conclusion, this study identifies CSF3R as a key driver of CD, linking its dysregulation to disease risk, immune dysfunction and inflammatory signaling. Its diagnostic accuracy and therapeutic potential highlight its clinical relevance, localizing CSF3R as a key gene and drug target. These findings provide a roadmap for future targeted therapeutic research aimed at modulating CSF3R activity and emphasize the importance of integrating genetic and molecular insights into clinical practice. Future studies should focus on validating these findings in larger cohorts, exploring the precise role of CSF3R in CD, advancing personalized CD diagnostic and therapeutic approaches, and translating these insights into clinical applications.

## Figures and Tables

**Table 1:** Basic information of datasets used in this study.

Dataset	Type	Platform	Sample	Biopsy tissue source
GSE112366	Microarrays	GPL13158	CD: 141 cases/control: 26 cases	Ileum tissue
GSE179285	Microarrays	GPL6480	CD: 47 cases (active)/control: 31 cases	Ileum tissue and colon tissue
GSE186582	Microarrays	GPL570	CD: 138 cases (active)/control: 25 cases	Ileum tissue
GSE75214	Microarrays	GPL6244	CD: 51 cases (active)/control: 11 cases	Ileum tissue
GSE193677	RNA-seq	GPL16791	CD: 160 cases/control: 120 cases	Ileum tissue
GSE119600	Microarrays	GPL10558	CD: 48 cases/control: 47 cases	Whole blood
K11_CD_STRICT2	FinnGen	–	CD: 2033 cases/control: 409940 cases	Ileum and colon tissue
GSE214695	ScRNA-Seq	GPL18573	CD: 6 cases (active)/control: none	Colon tissue

**Table 2:** The sequence of primers in *q*-PCR assay

Gene names	Sequences
CSF3R	F: CTCACAACCAGCAGCCTCATC
	R: GTCCTTGGGCACGCAGTC
β-Actin	F: TCCTTCCTGGGCATGGAGT
	R: AGCACTGTGTTGGCGTACAG

## Data Availability

Publicly available datasets were analyzed in this study. These data can be found inGEO, https://www.ncbi.nlm.nih.gov/geo/, GSE112366, GSE179285, GSE186582, GSE75214, GSE193677, GSE119600, and GSE214695, the IEU Open GWAS Project, https://gwas.mrcieu.ac.uk/, all eQTLs from GWAS Databases, and the FinnGen database, https://www.finngen.fi/en/, R10 K11_CD_STRICT2). R scripts for analyzing data are available on reasonable request.

## Supporting Information

Supporting Information 1 Supporting Information Table S1: Details of the 527 DEGs identified in the merged GSE112366, GSE179285, and GSE186582 dataset. Table S2: MR results of eQTL data with CD GWAS. Table S3: MR Results of the Intersected Genes. Table S4: Evaluation of machine learning diagnostic models for intersected genes_AUC Value. Table S5: GO enrichment analysis of Intersected genes. Table S6: KEGG enrichment analysis of Intersected genes. Table S7: GSEA enrichment analysis of Intersected genes. Table S8: Drugs targeting the CSF3R gene.

Supporting Information 2 Supporting Information Figure S1: Validation of CSF3R, GPR160, and RETSAT expression differences in tissue (Validation dataset GSE193677) and their diagnostic ROC curves. Figure S2: Validation of CSF3R expression in whole blood (Validation dataset GSE119600) and its diagnostic ROC curve.
