# Supplementary material for: Multiomics Integration and Machine Learning Reveal Colony Stimulating Factor 3 Receptor as a Key Gene and Therapeutic Target in Crohn's Disease
Source: Mediators Inflamm. 2025 Aug 28;2025:1619237. doi: 10.1155/mi/1619237 (PMC12411032; doi:10.1155/mi/1619237)
Supplement: Supporting Information 2 — Supporting Information Figure S1: Validation of CSF3R, GPR160, and RETSAT expression differences in tissue (Validation dataset GSE193677) and their diagnostic ROC curves. Figure S2: Validation of CSF3R expression in whole blood (Validation dataset GSE119600) and its diagnostic ROC curve. [file 1619237.f2.docx]

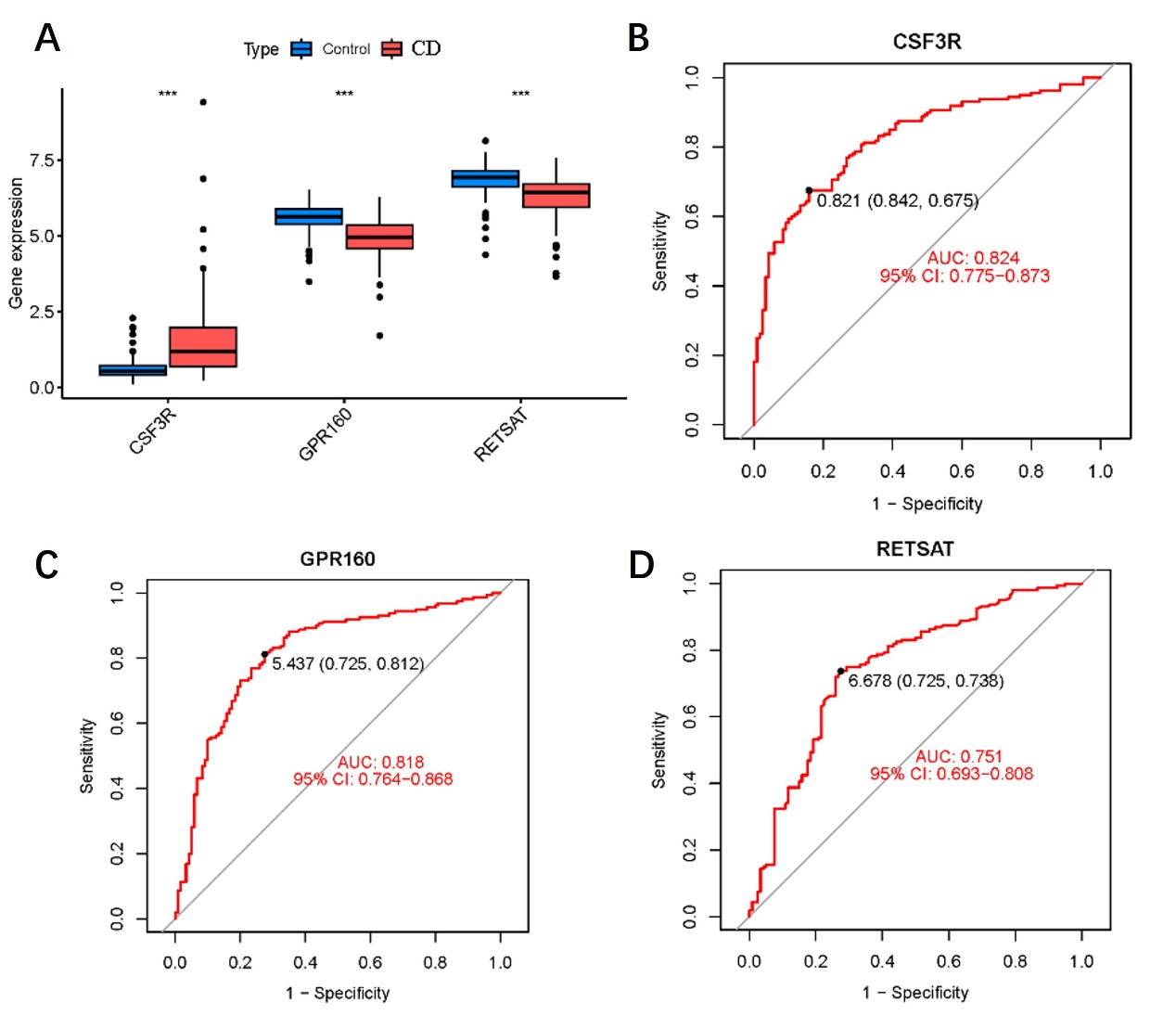


Fig.S1. (A) Relative expression levels of CSF3R, GPR160, and RETSAT in tissue samples from CD patients and healthy controls (Validation dataset GSE193677). The data demonstrate significantly higher expression levels of all three genes in the CD group compared to the healthy controls (***P<0.001). The box plots indicate the median, interquartile range, and data range. B–D: ROC evaluating the diagnostic performance of CSF3R (B), GPR160 (C), and RETSAT (D) expression levels for CD. Notably, CSF3R exhibits the largest AUC of 0.824 (95% CI: 0.775–0.873), indicating its superior sensitivity and specificity in distinguishing CD patients from healthy controls and suggesting its potential as a diagnostic biomarker for CD. GPR160 follows with an AUC of 0.818 (95% CI: 0.764–0.868). RETSAT also shows diagnostic potential with an AUC of 0.751 (95% CI: 0.693–0.808).


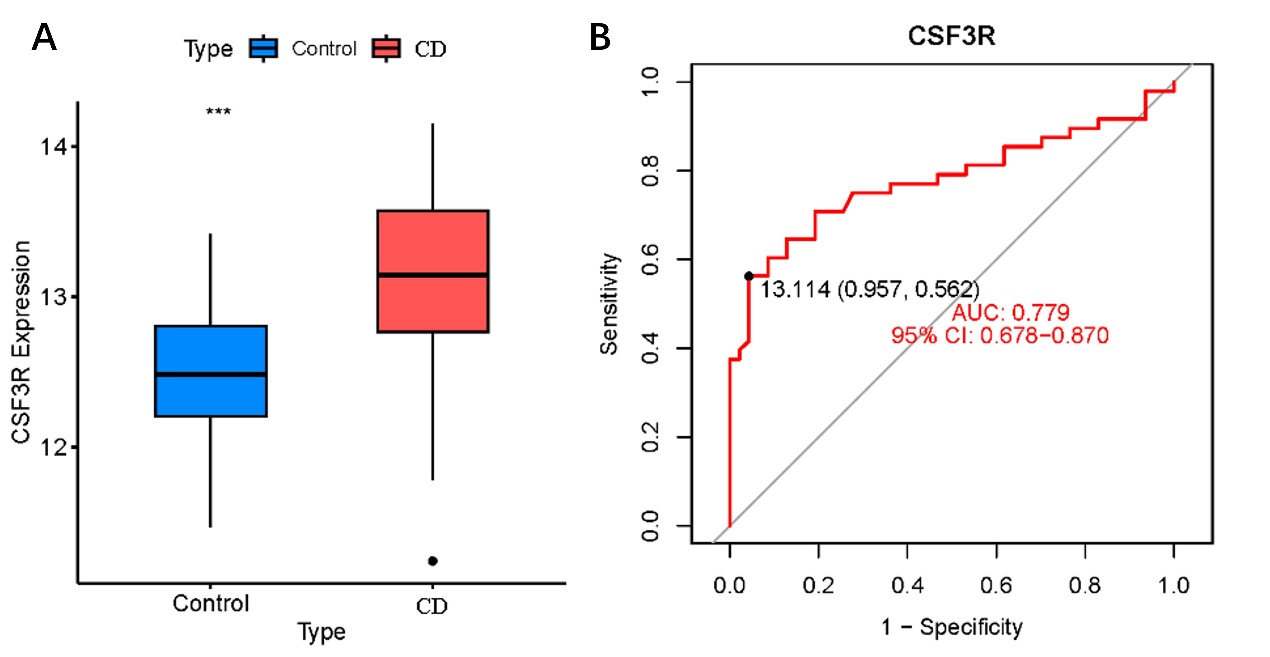


Fig.S2. (A) Differential expression of CSF3R in whole-blood samples of CD patients versus healthy controls (Validation dataset GSE119600). Box plots show significantly higher expression in CD patients (***P<0.001), with the center line, box, and whiskers representing the median, interquartile range, and data range. (B) ROC curve analysis of CSF3R expression for CD diagnosis. The AUC is 0.779 (95% CI: 0.678–0.870), indicating good diagnostic utility.
